# 

**Published:** 1921-09-24

**Authors:** 


### A Silver Wedding Gift.

Mr. Thomas R. Dootson, of Leigh, in Lancashire, celebrated his silver wedding by presenting the sum of £1,000 to the local town infirmary as a thankoffering for twenty-five years of happy married life.

### The Deaconess Hospital.

An interesting special hospital, for so it may be termed, is the Church of Scotland Deaconess Hospital (Lady Grisell Baillie Memorial). Opened in 1894, the patients comprise missionaries, deaconesses, and Church workers. There is an out-patient department; district visitors are attached, and the hospital treats children under the welfare scheme of Edinburgh Corporation. The Church of Scotland is much interested in the hospital and the Blantyre Mission, which form two of its most successful undertakings.

### An Unusual Source of Income.

The successful financial year of Bexley Cottage Hospital is partly due to the public spirit of a certain supplier who allowed the hospital "a large proportion of edibles at about 10 per cent. lower than the list price." Beside this, except for subscriptions and Hospital Sunday, most items of income showed a welcome increase. It is remarkable that in the thirty-seven years of the hospital's existence only two special appeals have had to be made. But future extensions must be prepared for, and then the committee will have to decide whether to build a new hospital rather than to spend money on alterations to the present building.

### Medical Certificate before Marriage?

In North Carolina a woman has been awarded $10,000 damages against her husband for infection with a venereal disease, and the Supreme Court has upheld the judgment. The United States Public Health Service points out the importance of this case, important because it sets aside in this particular case the legal barrier that prevents a wife from testifying against her husband and bringing suit against him. The fact that the North Carolina decision makes it likely that marriage will henceforth be no adequate defence against a suit for transmitting infection will probably hasten the adoption by the States of laws requiring every applicant for a marriage licence to present a certificate by a reputable doctor certifying that he is free from venereal disease, and providing that without this no licence shall be issued. Twenty States have already adopted laws forbidding persons with venereal disease to marry, amongst these are the States of New Hampshire, New Jersey, North Carolina, Oregon, Washington, and West Virginia. A similar Bill is now pending in Florida.

### The College of Nursing.

**(Limited by Guarantee.)**

### LONDON CENTRE.

Secretary, Miss M. A. Bompas, 7 Henrietta Street, W. 1.

A General Meeting of members of the London Centre will be held on Tuesday, October 4, at 6.30 p.m., at the College of Ambulance, 56 Queen Anne Street, W. 1. The matters to be discussed are important, and every member is asked to attend.

Members of the London Centre are reminded that the Jumble Sale will be held on October 15, and articles for the same can be sent to Miss Bompas, 7 Henrietta Street, W. 1.

### CORNWALL CENTRE.

(Hon. Secretary, Miss E. M. Jordan.)

There will be a meeting of members at the Royal Cornwall Infirmary, Truro, on September 27, at 3.30 p.m. It is hoped all will endeavour to be present.

### CARDIFF CENTRE.

(Hon. Secretary, Miss M. E. Hitch, 30 Newport Road.)

On Thursday last, September 15, some members of the Centre and their friends went by charabanc to visit the historic Tintern Abbey. The weather was ideal, and the scenery glorious, and it was agreed that it was an excellent way of spending a half-day.

### Matrons' and Sisters' Appointments.

London Jewish Hospital, Stepney Green.—Miss Lois Newman has been appointed matron. She was trained at the London Hospital, and has since been assistant matron at the Metropolitan Hospital, Kingsland Road; sister, out-patient sister, night superintendent, and temporary matron's office assistant at the London Hospital; matron of Guildford Military Hospital and acting matron of the Mines Hospital, Rio Tinto, Spain.

Eye Hospital, Bristol.—Miss Mallaley has been appointed sister. She was trained at Sheffield Royal Hospital, where she has been temporary sister.

Glasgow Royal Asylum, Gartnavel.—Miss Mary A. Lumsden has been appointed assistant matron. She was trained at St. George's Hospital, Hyde Park Corner, and at Crichton Royal Institution, Dumfries, and has since been sister at the West Herts Hospital, Hemel Hempstead.

Union Infirmary, South Shields.—Miss Edith J. Anderson has been appointed sister. She was trained at Bagthorpe Infirmary, Nottingham, and has since been ward sister. She has also been staff nurse at Liverpool, and health visitor and inspector of midwives to St. Helen's Corporation.

Union Infirmary, Hillingdon, Uxbridge.—Miss Louisa Wells has been appointed sister. She was trained at the City of Westminster Infirmary, Hendon, and has since been charge nurse to Berkhamstead Union Infirmary, and staff nurse at Winchmore Hill Isolation Hospital.

Royal West Sussex Hospital, Chichester.—Miss Louisa Edwards has been appointed assistant matron. She was trained at Stobhill Hospital, Glasgow, and has since been staff nurse at Woodside Hospital, Glasgow, and ward sister at the Royal West Sussex Hospital.

Mile End Hospital, Bancroft Road, Mile End.—Miss Mary Jane Eagle has been appointed sister-tutor and home sister. She was trained at Guy's Hospital, and has since been sister at Lewisham Hospital and has done private nursing.

Royal Liverpool Children's Hospital, Heswall.—Miss Beatrice Parker has been appointed night sister. She was trained at the Royal Southern Hospital, Liverpool, and Pendlebury Children's Hospital, and has since been sister of the children's ward at Harrogate Infirmary.

### St. Marylebone General Dispensary.

### POST-GRADUATE COURSE ON CHILD-WELFARE.

A course of twelve lectures on the Management and Feeding of Infants and Young Children will be given by Dr. Eric Pritchard (physician in charge of the Infant Welfare department) to qualified practitioners, commencing Wednesday, October 5, 1921, at the St. Marylebone General Dispensary (77 Welbeck Street, Cavendish Square, W. 1), at 6 o'clock in the evening.

Wednesday, October 5: "Causes of Infant Mortality."

Friday, October 7: "How to Conduct an Infant Consultation, Examine an Infant, and keep Records."

Wednesday, October 12: "Breast-feeding."

Friday, October 14: "The Artificial Feeding of Infants" (with special reference to Caloric Values, Balance Accessory Factors, etc.).

Wednesday, October 19: "The Modification of Milk."

Friday, October 21: "The Uses of Dried Milk and Patent Foods."

Wednesday, October 26: "The Management of Difficult Cases."

Friday, October 28: "Treatment of Minor Ailments."

Wednesday, November 2: "The Tuberculous Mother and her Child."

Friday, November 4: "The Syphilitic Mother and her Child."

Wednesday, November 9: "Rickets—Causes, Symptoms, and Treatment."

Friday, November 11: "The Feeding of Children between Nine Months and Two Years."

Students taking out this course will be entitled to attend the Infant Consultations held by Dr. Eric Pritchard on Tuesdays at 11 a.m., and Thursdays at 3 p.m., at the Dispensary (on these days practical demonstrations will be given as far as opportunity allows on the subjects referred to in the lectures. Opportunities will also be afforded to students of visiting the Nursery Training School, 1 Wellgarth Road, Golder's Green (five minutes from the Tube station), and of seeing the same methods employed there in dealing with resident infants. The visits will be paid on Saturday afternoons.

For tickets for the course apply to the Secretary, 77 Welbeck Street, Cavendish Square, W. 1.

**RATES OF SUBSCRIPTION** (post free, payable in advance).

Three months, 3/3; six months, 6/6; twelve months, 13/-. (Should the cost of printing and paper as well as increased postage, necessitate alteration of these rates, the period paid for will be reduced proportionately on unexpired subscriptions.) THE HOSPITAL "Index" to Volume LXIX., October 1920 to March 1921, is now ready, price 1/1 per copy, post free. Address The Manager, THE HOSPITAL, "The Hospital" Building, 28/29 Southampton Street, Strand, London, W.C. 2.

